# A mixed-methods approach to understanding pharmacists’ experiences with the CANRISK diabetes risk questionnaire and user guide

**DOI:** 10.24095/hpcdp.46.4.01

**Published:** 2026-04

**Authors:** Josephine Ta, Ying Jiang, Sebastian Srugo, Howard Morrison, Margaret de Groh

**Affiliations:** 1 Applied Research Division, Public Health Agency of Canada, Ottawa, Ontario, Canada; 2 Telfer School of Management, University of Ottawa, Ottawa, Ontario, Canada

**Keywords:** CANRISK, diabetes, pharmacists, CANRISK user guide, mixed-methods study

## Abstract

**Introduction:**

The Canadian Diabetes Risk Questionnaire (CANRISK) is a validated tool for diabetes risk screening, but the extent of its uptake and implementation by Canadian pharmacists has not been assessed. We aimed to describe the current use of the CANRISK tool and user guide among pharmacists, identify facilitators and barriers, and provide solutions to improve uptake.

**Methods:**

We used a mixed-methods approach comprising an initial quantitative online survey followed by qualitative interviews with pharmacists to allow for a deeper understanding of their experiences. Descriptive statistics were used to analyze the survey data and thematic analysis was used to analyze the interview data.

**Results:**

We found that 89% of pharmacists surveyed provided diabetes counselling on a daily or weekly basis, but more than half (55%) were not aware of CANRISK and its user guide. Of those who were aware, 60% indicated that they rarely or never used CANRISK. Five overarching themes were identified in the qualitative component. The facilitators to CANRISK uptake included pharmacist’s interest, diabetes clinic days/awareness campaigns, and patient-provider relationship. However, there are barriers to its implementation, including time constraints, competing priorities, financial pressures, staff shortages, and limited understanding of the tool’s usefulness.

**Conclusion:**

This paper found that CANRISK use was limited, and that support is needed to address the barriers for the successful implementation of CANRISK in pharmacies.

**Highlights**

- The Canadian Diabetes Risk Questionnaire (CANRISK) is a validated tool, but little is known about its implementation by pharmacists.
- This study identified some key facilitators to CANRISK implementation in the pharmacy setting, such as diabetes clinic days and patientprovider relationship. However, the tool is not widely used in pharmacies due to barriers such as competing priorities and limited understanding of its usefulness.
- Increasing awareness and understanding of the CANRISK tool, and providing more support are necessary to enhance the tool’s visibility and integration into routine practice

## Introduction

Diabetes is one of the most common chronic conditions in Canada and is increasing at a rate of 3.3% per year.^1^ Before diabetes develops, blood glucose levels rise above normal into the prediabetes range, a state that can be diagnosed and reversed if detected early. If left untreated, approximately 25% of people with prediabetes will progress to type 2 diabetes within 3 to 5 years, and up to 70% will develop diabetes within their lifetime.^2^ There is also evidence that preventing or delaying the onset of diabetes can reduce the risk of developing macrovascular complications such as cardiovascular disease, and microvascular complications such as retinopathy and nephropathy.^1^ Thus, early detection is key for better health outcomes. 

A valid and easily administered risk assessment tool, the Canadian Diabetes Risk Questionnaire (CANRISK: https://www.healthycanadians.gc.ca/en/canrisk), was developed by the Public Health Agency of Canada (PHAC) in 2011 to identify people at high risk of having prediabetes or diabetes.^3^ CANRISK scores are validated against reference standard blood tests and the questionnaire has been demonstrated to be a valid tool in Canada’s multi-ethnic population.^3^ Although developed over a decade ago, CANRISK remains a highly relevant and effective tool for diabetes risk assessment in Canada. Its continued evolution through ongoing validation efforts and targeted updates reinforces its viability. Recent initiatives, such as its adaptation for younger adults aged 18 to 39 and culturally tailored validation among African, Caribbean, and Black populations, reflect sustained investment and a strong commitment to health equity.^4^ When compared to similar tools like AUSDRISK and FINRISK, CANRISK performs comparably in identifying individuals at high risk of type 2 diabetes, making it a cost-effective and scalable solution.^3^

A previous evaluation of CANRISK from various regional public health perspectives supported its use as a diabetes risk screening and health promotion tool; however, the perspectives of pharmacists were not captured.^5^-^7^ Pharmacists play an important role in both diabetes prevention and management, and have led community-based diabetes screening programs.^8^-^17^ They are highly accessible in urban and rural areas, which place them in an ideal position to promote public health awareness and prevention of diabetes.^17^ CANRISK is a practical tool that can be implemented in pharmacies within primary care settings to enable early detection of individuals at risk for type 2 diabetes. The questionnaire contains simple items, such as age, waist circumference, family history, and physical activity level. It is also easy to administer, and its scoring system allows for quick identification of low-, moderate-, and high-risk individuals. In addition, CANRISK can be used to support both referral and counselling, where high-risk patients can be referred to primary care providers for confirmatory testing, and those with moderate risk can receive counselling from pharmacists on lifestyle interventions (diet, physical activity, and weight management). Similar tools such as AUSDRISK highlight the value of risk assessment screening. For example, the Pharmacy Diabetes Screening Trial (PDST) in Australia screened over 14 000 participants and identified 45% who required a referral.^18^ Following referrals, 136 participants were diagnosed with diabetes and 338 with prediabetes.^18^ Therefore, its integration in pharmacy workflows can facilitate early detection of at-risk individuals and allow pharmacists to play a key role in preventive care. The Canadian Pharmacists Association (CPhA) has also developed a CANRISK User Guide for Pharmacists, which provides supplementary information to facilitate CANRISK uptake.^19^-^20^ Still, little is known about pharmacists’ experience in implementing CANRISK. 

The objective of this study was to assess pharmacists’ experiences of the CANRISK tool and CPhA’s User Guide for Pharmacists, identify facilitators and barriers, and provide solutions to improve its uptake and use.

## Methods

***Study design***

This study used a mixed-methods approach involving an online survey and interviews with pharmacists. The sole inclusion criterion was licensed Canadian pharmacists in any practice setting.

***Data collection***

**Survey**

The survey was designed by a pharmacist who was also a Certified Diabetes Educator on the research team, and pretested by the full research team. Questions were also reviewed by Health Canada’s Public Opinion Research team. Available in both English and French, the survey contained 10 questions with a mix of binary, Likert scale, and open-ended questions to understand pharmacists’ experience and satisfaction with the tool. The survey was hosted on Qualtrics (Seattle, Washington, United States) from October 2022 to February 2023, and recruitment involved contacting organizations to help distribute the survey: CPhA (via their monthly newsletter in October and November 2022), Diabetes Canada (via their communications team), provincial pharmacists associations (only Pharmacists’ Association of Newfoundland and Labrador responded and they posted the survey link in their bi-weekly newsletter in November 2022), relevant stakeholders via Health Canada (HC) and PHAC’s Consultation and Stakeholder Information Management System, and previous pharmacists contacts. The snowball sampling method was also used to increase recruitment where previous contacts helped distribute the survey further through their professional networks. Snowball sampling was used because our target population comprises pharmacists across Canada in various practice settings, and because there is no exhaustive sampling frame of pharmacists actively performing diabetes risk assessment. This method leverages existing professional networks to increase response rates among busy clinicians, and has been used in prior studies involving pharmacists and diabetes education, and in diabetes risk research more broadly to recruit health care providers.^21^-^24^ We estimated that approximately 20 800 pharmacists received the survey link. The completion of the survey was voluntary. Participants were required to provide consent and were assured that all the information collected would be confidential. 

**Interviews**

At the end of the survey, participants were asked if they would like to share their experience on the CANRISK tool and/or user guide through participation in an interview. If yes, they were directed to provide their email address. They were then contacted by a member of the research team for a semi-structured interview. From December 2022 to February 2023, 20- to 30-minute one-on-one recorded interviews were conducted using Microsoft Teams (Redmond, WA, USA). Interview questions were developed and reviewed by the research team. Questions were also reviewed by Health Canada’s Public Opinion Research team. The interview was comprised of four to nine questions depending on the pharmacists’ experience and awareness of the CANRISK tool and/or user guide. If they were not aware of the CANRISK tool and/or user guide, the pharmacists would review them and provide their feedback. Interview questions can be found in the supplementary material (available on request from the authors). Interviews were recorded with permission from the participants. Verbal consent related to privacy and confidentiality was provided at the beginning of the interview. All participants accepted audio-recording, and consent was audio-recorded at the start of each interview. 

**Data analysis**

Descriptive statistics, including counts and percentages, were used to describe and summarize the survey data. Qualitative data from interviews were transcribed and analyzed thematically.^25^ First, a coding manual was developed based on the first two interviews by a research team member (JT). Another team member (SS) then coded these two interviews using the coding manual. Following this, the two team members (JT & SS) met to verify the congruence in code definitions and to refine and finalize the coding manual. They then independently coded the remaining five transcripts using the final coding manual. After the interviews were coded, the differences in coding were reviewed and discussed until consensus was achieved. Common categories, patterns, and concepts were identified, which led to themes. Data analysis (quantitative and qualitative) was completed on Microsoft Excel (Redmond, WA, USA). 

***Ethics approval***

Based on Article 2.5 of the Tri-Council Policy Statement: Ethical Conduct for Research Involving Humans, the Health Canada/PHAC Research Ethics Board concluded that ethics approval was not required.^26^ The Privacy Management Division at PHAC reviewed the project to ensure confidentiality and privacy guidelines were followed.

## Results

***Survey results***

A total of 44 respondents agreed to participate in the online survey, which demonstrated a low response rate (estimated < 1%) (Table 1). Among them, 89% provided daily to weekly prediabetes or diabetes counselling, but more than half (55%) of pharmacists were unaware of the CANRISK tool (Table 2). Of those who were aware, 60% indicated that they rarely or never used CANRISK. There was only one pharmacist who used another tool. In addition, 60% of the pharmacists who completed the survey were unaware of CPhA’s User Guide for Pharmacists. Those who were aware of it rarely or never used it. 

**Table 1:** Baseline characteristics of pharmacists (n = 44) in the survey

Characteristics	n	%
Years of experience		
0–5 years	4	9
6–10 years	15	34
11 years or more	25	57
Regions of practice^a^		
British Columbia	5	11
Prairies (AB, SK, MB)	1	2
Ontario	21	47
Quebec	15	33
Atlantic (NS, NB, NL, PEI)	3	7
Areas of primary practice setting^b^		
Medium-large population centre	39	89
Small-rural population centre	5	11

**Abbreviations: **AB, Alberta; MB, Manitoba; NB, New Brunswick; NL, Newfoundland and Labrador; NS, Nova Scotia; PEI, Prince Edward Island; SK, Saskatchewan. 

^a ^No responses from the Territories. 

^b^ Medium-large population centre is defined as population between 30 000 and 100 000 or more. Small-rural population centre is defined as population between < 1000 and 29 999. 

**Table 2:** Survey questions and results

Questions	n	%
How many patients do you see in a typical week? Include all patients, including those who visit you for diabetes and non-diabetes related visits.		
Less than 20	2	5
20 to 50	6	14
51 to 80	2	5
81 to 110	11	25
111 to 140	5	11
Over 140	18	41
On average, how often do you provide prediabetes or diabetes advice to your patients?		
Daily	28	64
Weekly	11	25
Monthly	0	0
A few times per year	3	7
Less than a few times a year	2	5
Never	0	0
Are you aware of the CANRISK tool?		
Yes	20	45
No	24	55
i) [If replied “Yes”] How did you find out about CANRISK?		
From pharmacy school	8	40
At a conference	3	15
From work	7	35
From a colleague	0	0
Others (specified email, literature, guidelines)	2	10
ii) [If replied “Yes”] How often do you use the CANRISK questionnaire for prediabetes and diabetes risk assessment?		
Daily	0	0
Weekly	0	0
Monthly	2	10
A few times per year	6	30
Less than a few times a year	6	30
Never	6	30
Are you using other tools for prediabetes and diabetes risk assessment?		
Yes (participant specified CDPP)	1	2
No	43	98
Are you aware of the CPhA’s user guide for CANRISK?		
Yes	8	40
No	12	60
i) [If replied “Yes”] How frequently do you consult the user guide while implementing the CANRISK tool for risk assessment?		
Daily	0	0
Weekly	0	0
Monthly	1	13
A few times per year	2	25
Less than a few times a year	2	25
Never	3	38
ii) [If replied “Yes”] How would you rate how easy it is to use the user guide?		
Difficult	0	0
Hard	0	0
Average	3	38
Easy	5	63
Very easy	0	0
Do you think a similar guide for the general public or community workers helping others use the CANRISK tool in a community setting would be helpful?		
Yes	18	90
No	2	10

**Abbreviations:** CANRISK, Canadian Diabetes Risk Questionnaire; CDPP, Canadian Diabetes Prevention Program; CPhA, Canadian Pharmacists Association. 

**Notes: **Percentages are based on the number of respondents for each subquestion. Percentages may not add up to 100 due to rounding. 

***Interview findings***

Seven pharmacists were interviewed: five from Ontario, one from the Atlantic Region, and one from Quebec. Concepts sorted by themes are found in Table 3. Data saturation was assumed to be achieved as the final three participants did not present any additional themes. Five themes emerged and are described as follows.

**Table 3:** Occurrence of concepts in the interviews (n = 7)

Concepts	Occurrence, # of pharmacists
Theme 1: Pharmacist’s role and interest	
Pharmacist has a role in identifying at-risk patients	6/7
Pharmacist with personal interest in diabetes or pharmacist who is also a CDE is more likely to use CANRISK	4/7
Good patient-provider relationship	5/7
Pharmacists are first point of contact to healthcare system	5/7
Theme 2: Positive perception of CANRISK	
Tool is useful	5/7
Used/can be used in diabetes clinic days/awareness campaigns	5/7
Theme 3: Limited use of the user guide	
Awareness of user guide	4/7
Have used the user guide in practice	1/4
Positive feedback after reviewing user guide for those who are not aware of it	3/3
Theme 4: Barriers to implementation	
Time constraints	7/7
Competing priorities	7/7
Financial pressures	6/7
Staff shortages	5/7
Limited understanding of the usefulness of CANRISK	4/7
Theme 5: Recommendations	
Increasing awareness/uptake	
Education to increase CANRISK uptake	5/7
Put posters in pharmacies to increase awareness	3/7
Develop webinars on CANRISK	2/7
Use user guide to create webinar content	3/7
Improving CANRISK tool	
Improve readability	4/7
Avoid potentially offensive language	5/7
Provide explanation on sex vs. gender	4/7
Improve procedure to measure waist circumference in public setting	5/7
Provide information for next steps after patient completes the questionnaire	7/7
Improving CANRISK user guide	
Update the content (outdated)	4/7
Develop webinar with content in user guide	3/7

**Abbreviations:** CANRISK, Canadian Diabetes Risk Questionnaire; CDE, Certified Diabetes Educator.

**Theme 1: Pharmacist’s role and interest**

Most interviewees highlighted that pharmacists could play a key role in identifying individuals at risk of type 2 diabetes due to their accessibility and expertise. Using CANRISK, pharmacists can screen and educate patients about modifiable risk factors such as diet, physical activity, and weight management. They can also refer high-risk individuals to primary care providers for confirmatory testing, and provide ongoing support and follow-up. Therefore, there is an opportunity to incorporate CANRISK into pharmacy services to facilitate early detection and raise public awareness.

Interviewees noted that, based on their clinical experience and/or observations, some pharmacists who have an interest in diabetes and/or are Certified Diabetes Educators (CDEs) tend to include CANRISK in their discussions with patients and in diabetes clinic days/awareness campaigns. Meanwhile, in terms of uptake, they noted that patients are more likely to complete the questionnaire if there is a good patient-provider relationship, or if the patient does not have a primary care physician, as one pharmacist mentioned: “(When) patients themselves sense something is wrong, they are more likely to come to a pharmacist that they know personally to seek help.” (Pharmacist 2)

**Theme 2: Positive perception of CANRISK**

The pharmacists, who are using CANRISK in their practice, found the tool to be helpful in identifying at-risk patients, and would usually incorporate CANRISK in their diabetes clinic days or awareness campaigns. Most pharmacists (5 out of 7 pharmacists) support its use, as shown by one of their comments: “It’s a very good basic tool actually, that is underutilized, very underutilized.” (Pharmacist 3)

**Theme 3: Limited use of the user guide**

Some pharmacists were aware of the CPhA’s User Guide for Pharmacists, but only one pharmacist from the interview consulted it in practice. When the user guide was reviewed by the pharmacists who were not aware of it, they found it to be informative and a good complement to the questionnaire. “Yeah, I’m not familiar with the user guide.” (After reviewing:) “Oh, this is good! It’s good. Yeah, it has a lot of details and everything.” (Pharmacist 2)

**Theme 4: Barriers to implementation**

Challenges included time constraints, competing priorities, financial pressures, staff shortages, and limited understanding of the usefulness of CANRISK. Education about CANRISK was highlighted by several pharmacists as a solution to increase its uptake. “The other part is just awareness. Patients aren’t aware that this exists. Lots of health professionals aren’t aware that this exists.” (Pharmacist 1)

**Theme 5: Recommendations**

The pharmacists who have used CANRISK provided recommendations based on their experience, and those who have not used it reviewed the questionnaire and the user guide and offered suggestions to improve them. Recommendations regarding how to increase CANRISK awareness and uptake included putting posters in pharmacies to increase patient conversations, developing webinars on CANRISK for pharmacists, and possibly using the user guide to create the webinar content. This could help increase awareness and help pharmacists feel more confident when administering the questionnaire. 

Recommendations to improve the questionnaire included improving readability (larger font size, simpler words), avoiding potentially offensive language (avoid the word “large” when referring to weight, using a preamble to provide a rationale regarding sex vs. gender), improving procedures for measuring waist circumference in a public setting, and providing options on next steps once an individual has received a high-risk score. For example, next steps could include asking the patient to speak to a health care provider for bloodwork to assess risk, and providing a pamphlet/information sheet to the patient after they complete the questionnaire to put the risk into context. 

Some recommendations for the user guide include updating the content as some of the information and links are outdated and using the content of the user guide to develop a webinar or continuing education material for pharmacists. 

## Discussion

This is the first study that examined pharmacists’ experiences with the CANRISK tool and user guide. The survey results revealed that most pharmacists were unaware of CANRISK and its use in pharmacies was limited. The themes identified in the interviews helped to explain the barriers to CANRISK implementation, but also highlighted that there is potential to improve CANRISK uptake in pharmacy settings. 

Our findings illustrated that the uptake of CANRISK by pharmacists is limited, which is consistent with the systematic review that examines health care professionals’ uptake of diabetes risk assessment tools.^27^ Yet, the findings from Bird and colleagues, which examined the use of CANRISK from the perspectives of regional public health organizations, found that the tool was widely used for risk screening and health promotion.^5^ However, they sampled mostly administrators of health organizations as compared to the front-line health care professionals targeted in our study.^5^

The interviews identified barriers to implementing CANRISK, such as time constraints, competing priorities, financial limitations, staff shortages, lack of awareness, and limited understanding of its utility. These barriers align with findings from a systematic review and existing literature, which include attitudes toward diabetes assessment tools, impracticality of the tools, and lack of reimbursement and regulatory support.^27^,^28^ The lack of CANRISK awareness may stem from minimal promotion since CANRISK’s launch over a decade ago. When CANRISK was first launched, CPhA developed the CANRISK User Guide for Pharmacists, which provides supplementary information, key messages, and clarification that pharmacists can provide to patients. As with many programs, uptake often peaks early and then declines, which may explain the current limited use. Our results found that although more than half of the surveyed pharmacists were unaware of CANRISK, those who used it incorporated it into their diabetes awareness efforts. This suggests that there are opportunities to engage pharmacists more effectively to assist in early identification of individuals at risk of developing type 2 diabetes and to refer them to primary care providers promptly. Pharmacists are increasingly playing a key role in the prevention and management of chronic diseases, such as diabetes, as seen in several pharmacist-led interventions in the literature.^27^,^29^-^34^ In particular, community pharmacists are in a unique position to support CANRISK implementation due to their accessibility, regular interactions with patients, and expertise in chronic disease prevention and management. While staffing shortages and competing priorities are some of the challenges, CANRISK could be integrated into existing activities, such as patient counselling or medication reviews, as an opportunistic risk screening. By way of illustration—pending feasibility testing—while patients wait for their prescriptions or flu shots, a pharmacy assistant or technician could introduce the CANRISK questionnaire to them. Patients could complete it on paper or a tablet, after which the pharmacist would review the results, provide counselling, and, if needed, refer patients to a physician. Results could then be documented in the pharmacy system for follow-up.

The pharmacists in the interview also provided recommendations on the CANRISK tool, which are consistent with the systematic review and Bird et al’s study.^5^,^27^ Recommendations include improving readability, addressing potentially offensive language, improving procedures for measuring waist circumference in public settings, and providing clear next steps for individuals with high-risk scores.^5^,^27^ The similarity in recommendations on CANRISK by pharmacists and other audiences indicates that the CANRISK tool may need to be revised to enhance its usability and relevance. 

***Strengths and limitations***

This study provides valuable insights from pharmacists on their use of CANRISK and the user guide. As pharmacists are highly accessible with an expanded scope of practice (which varies across jurisdictions), a better understanding of what will support their use is important. Limitations of this study include small sample sizes, certain provinces not being represented and others overrepresented (e.g. Ontario and Quebec), and the non-random method of obtaining respondents (i.e. snowball sampling), which limit its generalizability. Although various organizations and previous pharmacist contacts helped to distribute the survey, a low response rate was found. Some factors that may contribute to the low response include time constraints, lack of incentives for participating in the survey, and survey fatigue (if they are invited to participate in multiple surveys), which are consistent with the literature.^35^ Low participation may also suggest limited interest, awareness, or time for CANRISK. Meanwhile, the pharmacists who participated might already have an interest in diabetes prevention, which may impact our findings through selection bias. Despite the limitations, this study informs current use and future evaluation of CANRISK. 

## Conclusion

CANRISK remains a relevant and valuable tool for early identification of type 2 diabetes risk, with recent updates expanding its use to younger and more diverse populations. It serves not only as a no/low-cost screening instrument but also as an effective referral and counselling aid in pharmacy settings, supporting timely intervention and health education. Our findings suggest that more support is needed to address the barriers of CANRISK implementation in pharmacy settings and a revision of the tool is recommended, specifically improving readability, avoiding potentially offensive language, and providing information on next steps after patients complete the questionnaire. Further studies are needed to evaluate the impact of CANRISK implementation in pharmacy settings and potential cost savings to the health care system due to pharmacists’ intervention, such as referral to primary care providers, drug therapy recommendations, and lifestyle interventions. 

## Acknowledgements

The authors express their thanks and appreciation to the Canadian Pharmacists Association (CPhA), Diabetes Canada, Pharmacists’ Association of Newfoundland and Labrador, and previous contacts for assisting in distributing the survey. We also express our thanks to the pharmacists who participated in the survey and those who agreed to take part in the interviews and shared their experiences and insights regarding the CANRISK tool and the user guide. This study was made possible by the internal research capacity of the Public Health Agency of Canada. 

## Conflicts of interest

Margaret de Groh is the journal’s former Associate Editor-in-Chief and Howard Morrison is an Acting Editor-in-Chief and one of the journal’s Editorial Board Members. Both have recused themselves from the review process for this article. The authors declare that they have no competing interests.

## Authors’ contributions and statement

JT: conceptualization, formal analysis, writing—original draft, writing—review and editing. 

YJ: conceptualization, writing—review and editing, supervision.

SS: formal analysis, writing—review and editing.

HM: writing—review and editing.

MdG: conceptualization, writing—review and editing, supervision.

The content and views expressed in this article are those of the authors and do not necessarily reflect those of the Government of Canada.
